# A Case of Constant and Protracted Vomiting from Pregnancy

**Published:** 1879-08

**Authors:** H. L. Wilson

**Affiliations:** Atlanta, Georgia


### A CASE OF CONSTANT AND PROTRACTED VOMITING FROM PREGNANCY.

By H. L. WILSON, M.D., Atlanta, Georgia.

June 23d I was called to see Mrs. G., a blonde, some 20 years of age, tolerably stout, whose general health was good. I found her suffering greatly from nausea; she could not turn or assume the erect posture without its producing emesis. While she had an apetite it was impossible for her to retain nourishment long enough to be benefitted or supported by the same, hence she was daily growing weaker,

I learned from Mrs. G. that during a previous pregnancy, in the spring of 1878, she was confined to her bed during the entire term, and that nothing seemed to be of any avail that was administered. In fact, she came near dying from inanition, and was advised by her physicians that a second impregnation might terminate fatally. However, she did go the full time, and was delivered of a healthy child.

When I saw her, June 23d, she was only pregnant about six weeks, and the sickness had then confined her to her bed about three weeks. I found her lying upon her back, the only position she could take, her skin cold and clammy, hot flushes occasionally, eyes sunken and uneasy-looking. Even the effort to talk caused vomiting. The muscles of the abdomen were quite sore from the constant effort. I ordered a large mustard plaster put over the lower portion of the bowels, and gave minute doses of hy. chlo. mite. every three hours, until catharsis was produced. In the meantime, I administered iced mint julep, camphor water, etc. I ordered lime water and sweet milk as a nourishment, but she could not retain it but a few moments. I gave ingluvin, grs. v, every four hours for twenty-four hours. I followed this with oxalate of cerium in doses of two grs., with no benefit to my patient. I then resorted to dry cups over the lower portion of the back, giving lactopepsin internally. By this time the counter-irritation over the abdomen was very thorough, but I felt my patient was not improving. The husband was inclined to have abortion produced, believing from last year's experience that she could not survive it. I urged him to be patient, and gave Mrs. G. a hypodermic injection of morphine containing gr. $\frac{1}{3}$. This gave her a good nights sleep, which rested and revived her a great deal. I repeated the same every twelve hours for three days when it became apparent that she could not be carried any longer on that line. I informed her husband that I desired to make a direct application to the mouth of the womb, hoping thereby, to relieve the irritation. He consented, and on the morning of July 5, 1879, I made an application of nitrate of silver, 40 grs. to the ounce, directly over the os-tincae. The next day she seemed a little more quiet, but having withdrawn the hypodermic injection of morphine, I inferred that possibly, some of the nausea was caused by the want of it. I now gave iced champagne which was discontinued on the 3d day, she feeling that she was receiving no benefit. She was now partly nourished by enemas, but occasionally took sweet milk or gruel, per orem, but rejected it in a few moments. During all this time, I could not induce her to remain in any position save upon the back.

As she was now on ingluvar again and cracked ice, I determined to make another application of nitrate of silver, wich I did July 9th, making a very thorough touch over the mouth and neck of the womb. The next day she was more comfortable and retained some nourishment.

July 11—Improving steadily.

July 13—Was able to sit up in rocker.

July 15—Found my patient absolutely using sewing machine, which I positively forbade. By this time she only had nausea occasionally with vomiting in the mornings, but is doing as well as could be expected.

Up to this date, I have not been called to renew the application. I am satisfied that in these very extreme cases, there is no remedy equal to the nitrate of silver; but for its use, abortion would have probably been necessary to save the life of my patient.

